# COVID‐19 vaccine effectiveness against hospitalization due to SARS‐CoV‐2: A test‐negative design study based on Severe Acute Respiratory Infection (SARI) sentinel surveillance in Spain

**DOI:** 10.1111/irv.13026

**Published:** 2022-07-26

**Authors:** Clara Mazagatos, Concepción Delgado‐Sanz, Susana Monge, Francisco Pozo, Jesús Oliva, Virginia Sandonis, Ana Gandarillas, Carmen Quiñones‐Rubio, Cristina Ruiz‐Sopeña, Virtudes Gallardo‐García, Luca Basile, María Isabel Barranco‐Boada, Olga Hidalgo‐Pardo, Olalla Vazquez‐Cancela, Miriam García‐Vázquez, Amelia Fernández‐Sierra, Ana Milagro‐Beamonte, María Ordobás, Eva Martínez‐Ochoa, Socorro Fernández‐Arribas, Nicola Lorusso, Ana Martínez, Ana García‐Fulgueiras, Bartolomé Sastre‐Palou, Isabel Losada‐Castillo, Silvia Martínez‐Cuenca, Mar Rodríguez‐del Águila, Miriam Latorre, Amparo Larrauri, The SARI surveillance VE group in Spain

**Affiliations:** ^1^ National Centre for Epidemiology Institute of Health Carlos III Madrid Spain; ^2^ Consortium for Biomedical Research in Epidemiology and Public Health (CIBERESP) Madrid Spain; ^3^ Consortium for Biomedical Research in Infectious Diseases (CIBERINFEC) Madrid Spain; ^4^ National Centre for Microbiology Institute of Health Carlos III Madrid Spain; ^5^ Subdirección General de Epidemiología Dirección General de Salud Pública Madrid Spain; ^6^ Servicio de Epidemiología y Prevención Sanitaria Dirección General de Salud Pública, Consumo y Cuidados Logroño Spain; ^7^ Dirección General de Salud Pública Junta de Castilla y León Valladolid Spain; ^8^ Dirección General de Salud Pública y Ordenación Farmacéutica Junta de Andalucía Seville Spain; ^9^ Subdirección General de Vigilancia y Respuesta a Emergencias de Salud Pública Agencia de Salud Pública Catalonia Spain; ^10^ Servicio de Epidemiología Dirección General de Salud Pública, Consejería de Salud Murcia Spain; ^11^ Servicio de Medicina Preventiva Hospital Universitario Son Espases Servicio de Epidemiología, Consellería de Salut Palma Spain; ^12^ Servicio de Medicina Preventiva Complejo Hospitalario Universitario de Santiago Santiago de Compostela Spain; ^13^ Vigilancia Epidemiológica, Dirección General de Salud Pública, Departamento de Sanidad Gobierno de Aragón Zaragoza Spain; ^14^ Servicio Medicina Preventiva Hospital Universitario Virgen de las Nieves Granada Spain; ^15^ Laboratorio de Microbiología Hospital Universitario Miguel Servet Zaragoza Spain; ^16^ Instituto de Investigación Sanitaria Aragón (IIS Aragón) Zaragoza Spain; ^17^ Servizo de Epidemioloxía, Dirección Xeral de Saúde Pública, Consellería de Sanidade Xunta de Galicia Galicia Spain

**Keywords:** COVID‐19, COVID‐19 vaccine, SARI surveillance, SARS‐CoV‐2, Spain, test‐negative design, vaccine effectiveness

## Abstract

**Background:**

With the emergence of SARS‐CoV‐2, influenza surveillance systems in Spain were transformed into a new syndromic sentinel surveillance system. The Acute Respiratory Infection Surveillance System (SiVIRA in Spanish) is based on a sentinel network for acute respiratory infection (ARI) surveillance in primary care and a network of sentinel hospitals for severe ARI (SARI) surveillance in hospitals.

**Methods:**

Using a test‐negative design and data from SARI admissions notified to SiVIRA between January 1 and October 3, 2021, we estimated COVID‐19 vaccine effectiveness (VE) against hospitalization, by age group, vaccine type, time since vaccination, and SARS‐CoV‐2 variant.

**Results:**

VE was 89% (95% CI: 83–93) against COVID‐19 hospitalization overall in persons aged 20 years and older. VE was higher for mRNA vaccines, and lower for those aged 80 years and older, with a decrease in protection beyond 3 months of completing vaccination, and a further decrease after 5 months. We found no differences between periods with circulation of Alpha or Delta SARS‐CoV‐2 variants, although variant‐specific VE was slightly higher against Alpha.

**Conclusions:**

The SiVIRA sentinel hospital surveillance network in Spain was able to describe clinical and epidemiological characteristics of SARI hospitalizations and provide estimates of COVID‐19 VE in the population under surveillance. Our estimates add to evidence of high effectiveness of mRNA vaccines against severe COVID‐19 and waning of protection with time since vaccination in those aged 80 or older. No substantial differences were observed between SARS‐CoV‐2 variants (Alpha vs. Delta).

## 1 INTRODUCTION

The emergence of the severe acute respiratory syndrome coronavirus 2 (SARS‐CoV‐2) in late 2019 and the following coronavirus disease (COVID‐19) pandemic had a great impact on influenza surveillance systems.^1^ In Spain, influenza surveillance before the COVID‐19 pandemic was based on a sentinel network of primary care physicians as well as a hospital network.^2^ When SARS‐CoV‐2 emerged in 2020, the hospital‐based system, which involved the reporting of confirmed influenza cases, was unable to detect early COVID‐19 hospitalizations. In addition, the creation of SARS‐CoV‐2 testing centers outside of the usual primary care circuits and the reallocation of sentinel physicians to other centers led to the disruption of the sentinel primary care influenza surveillance network for the first time since it was established in 1996.^3^

The World Health Organization (WHO) and the European Centre for Disease Prevention and Control (ECDC) recommended that countries adapt their existing influenza surveillance systems and supported the implementation of sentinel systems for the syndromic surveillance of respiratory viruses, including influenza and SARS‐CoV‐2.^4^, ^5^ Following these recommendations, the Acute Respiratory Infection Surveillance System (SiVIRA in Spanish) was created in Spain in 2020. It incorporates a sentinel network for acute respiratory infection (ARI) surveillance in Primary care and a network of sentinel hospitals for Severe ARI (SARI) surveillance. The Horizon2020 I‐MOVE‐COVID‐19 “Multidisciplinary European network for research, prevention and control of the COVID‐19 Pandemic,” launched in March 2020 with the objectives of reinforcing the surveillance of COVID‐19 and studying associated risk factors and COVID‐19 vaccine effectiveness (VE) in European countries, served as a pilot experience for SARI surveillance in Spain. The collaboration of the hospitals involved in I‐MOVE‐COVID‐19, with previous experience in influenza VE studies as well, was an essential guide for other hospitals and regions in the design and subsequent implementation of SARI surveillance in Spain. ECDC has also supported the creation of a European SARI surveillance network (E‐SARI‐NET) and multicountry COVID‐19 VE studies in Europe. The first VE estimates against SARI associated with laboratory‐confirmed SARS‐CoV‐2 were published in October 2021.^6^ SARI surveillance was successfully implemented in 9 of 19 Spanish regions, with a total of 13 sentinel hospitals included in the national network during the 2020/21 SiVIRA pilot season.^7^

COVID‐19 vaccination in Spain began on December 27, 2020, initially prioritizing long‐term care facilities and health care workers and progressively extending to the general population. By October 3, 2021, 77.6% of the total Spanish population was fully vaccinated.^8^ Monitoring the real‐world effectiveness of COVID‐19 vaccines is essential to guide public health action and decision making, and even more so in a dynamic pandemic context with emerging new challenges such as new SARS‐CoV‐2 variants, or waning immunity. With case‐based data on a representative sample of SARI admissions, the SiVIRA surveillance system constitutes an appropriate platform for responding to these emerging challenges and for timely measuring of VE against severe forms of COVID‐19.

The aim of this study was to provide COVID‐19 VE estimates against COVID‐19 hospitalization, by age group, type of vaccine, time since vaccination, and SARS‐CoV‐2 variant, using a test‐negative design. As a data source, we used the information obtained from the SARI sentinel surveillance during season 2020–2021, the first season in which SiVIRA was implemented in Spain.

## 2 METHODS

### 2.1 Study design

A total of 13 sentinel hospitals from nine Spanish regions participated in the SARI surveillance. As described in the surveillance protocol,^9^ case‐based data were collected for a systematic weekly sample of patients who were hospitalized on Tuesday and/or Wednesday, depending on the Spanish region, meeting the SARI case definition (Appendix 1 in the Supporting Information). Patients were swabbed for RT‐PCR or rapid antigen test for SARS‐CoV‐2, and demographic, clinical, and COVID‐19 vaccination data were collected from hospital records and vaccine registries. We used a test‐negative case–control study design to estimate COVID‐19 VE.^10^

Where feasible, SARS‐CoV‐2 viruses from RT‐PCR positive cases were sequenced, and phylogenetic analysis was performed to identify the SARS‐CoV‐2 variant and lineage. If available, sequencing results were linked with epidemiological and clinical data merging through a unique sample identifier.

### 2.2 Study period

The study period included data reported between Weeks 53/2020 and 39/2021, including SARI patients with swab dates between January 1, 2021, and October 3, 2021.

### 2.3 Study inclusion criteria

**FIGURE 1:**
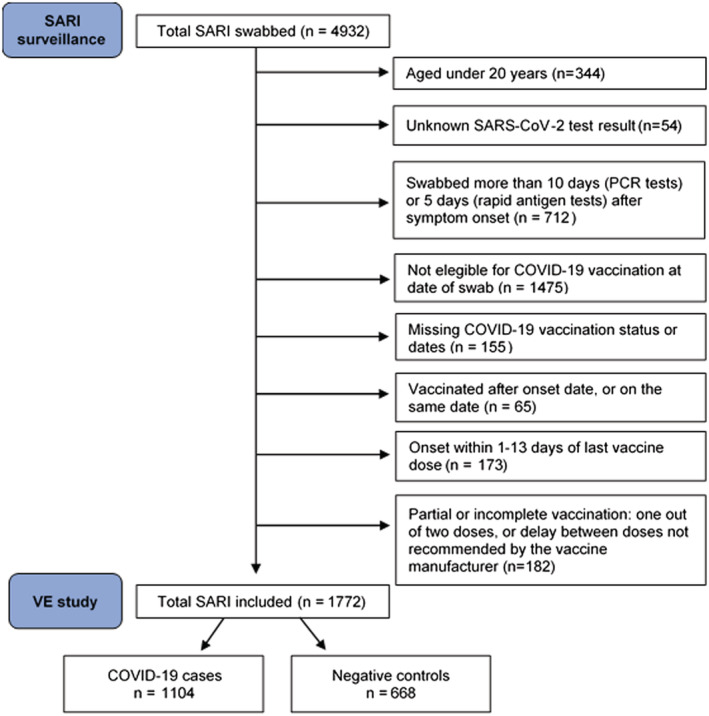
Flowchart for SARI patient inclusion in the VE study, Spanish SARI sentinel surveillance, Weeks 1–39/2021

We included patients aged 20 years and older who were part of an age‐specific target group for vaccination at time of swab (Table S1), with positive or negative SARS‐CoV‐2 results and known COVID‐19 vaccination status. We excluded those who were swabbed more than 10 days (RT‐PCR tests) or 5 days (rapid antigen tests) after symptom onset. We excluded patients vaccinated with first dose on or after onset date and those who did not meet the complete vaccination schedule in terms of number of doses and delay between doses. We further excluded those with symptom onset within 1–13 days of latest dose of COVID‐19 vaccine (Figure 1).

### 2.4 Case definitions

Cases and controls were defined as SARI patients testing positive or negative for SARS‐CoV‐2 in an RT‐PCR or rapid test, in the first 10 or 5 days, respectively, since symptom onset. There were no influenza positive cases among SARS‐CoV‐2 negative controls.

In the variant‐specific analyses, a case was defined as RT‐PCR positive with SARS‐CoV‐2 Alpha (B.1.1.7 lineage) or Delta variants (B.1.617.2 or AY lineages) confirmed through next generation sequencing (NGS). We only used negative controls from weeks with sequenced cases, removing all controls from weeks before and after the first and last case. We also removed controls from hospitals with no reported sequencing information.

We classified eligible cases and controls as either completely vaccinated or unvaccinated, dropping those with partial vaccination schedules. Definitions used for complete vaccination can be found on Appendix 1 of Supporting Information.

### 2.5 Statistical analysis

We compared the odds of complete COVID‐19 vaccination between cases and controls using a logistic regression, and VE was estimated as 1‐OR. We adjusted for age, sex, and presence of at least one chronic condition (hypertension, heart disease, chronic respiratory disease, diabetes, liver disease, renal disease, immunodeficiency or other chronic conditions). Age was modeled as restricted cubic splines (RCS), and swab date was modeled as RCS or month of swab, depending on the analysis. For the age‐specific analyses, we stratified the data into the following age groups: 20–39, 40–59, 60–69, 70–79, and ≥80 years. For some analyses, we stratified age into wider groups to increase sample size.

We measured VE, overall, and for mRNA vaccines, by time between vaccination and onset of symptoms with cut‐off points stratified every 3 months: <90, 90–150, and ≥150 days between last vaccine dose and symptom onset. All analyses were conducted using Stata version 16.1 (StataCorp, College Station, Texas 77845, USA).

### 2.6 Informed consent

All data used for this study were collected as part of routine surveillance, and informed consent or official ethical approval was not required, as regulated by Royal Decree 2210/1995 of December 28 provided by the Ministry of Health and Consumer Affairs. Although individual informed consent was not required, all data were pseudoanonymised to protect patient privacy and confidentiality.

## 3 RESULTS

### 3.1 Characteristics of cases and controls

**FIGURE 2:**
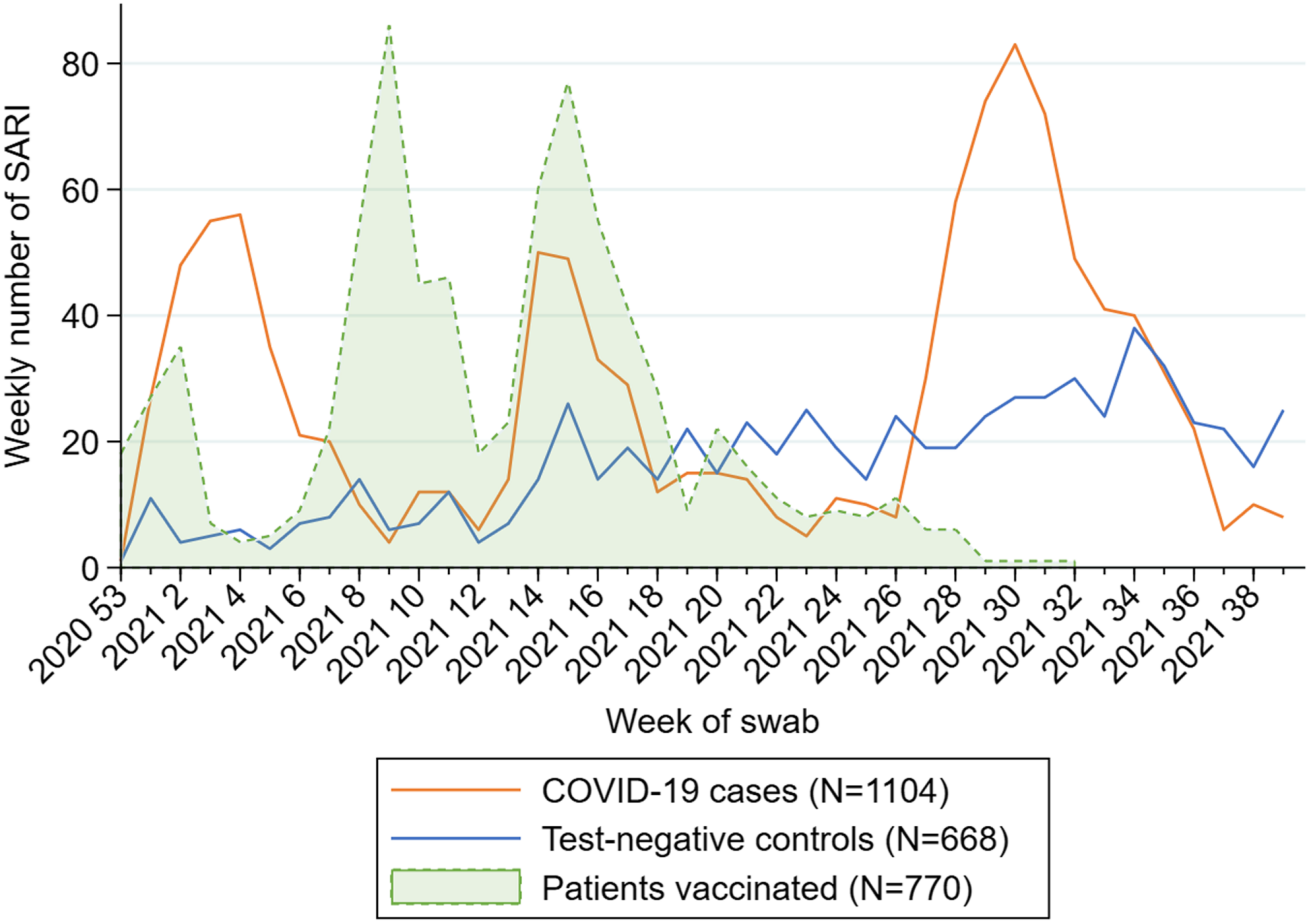
Number of SARI COVID‐19 cases and negative controls by week of swab and number of SARI vaccinated by week of complete vaccination, Spanish SARI sentinel surveillance, Weeks 1–39/2021

We included 1772 SARI patients aged 20 and older, of which 1104 were positive to SARS‐CoV‐2 (cases) and 668 were negative (controls) (Figure 1). Among the cases and controls, 770 (43%) had received complete COVID‐19 vaccination at least 14 days before symptom onset (Figure 2).

More than 75% of controls and 58% of cases were aged 70 and older, and the median age was 81 for controls and 71 for cases. The prevalence of underlying conditions was higher among controls than cases, with significant differences for all chronic conditions, except for chronic liver disease. Clinical presentation was more severe among cases than controls: 85% cases versus 51% controls had pneumonia, and 8% cases versus 1% controls required mechanical ventilation support. Cases had significantly higher proportion of ICU admission (10% vs. 2%) and death (18% vs. 10%) than controls (Table 1).

**TABLE 1:** Characteristics of SARI controls and cases (n = 1772) recruited for the VE study, Spanish SARI sentinel surveillance, Weeks 1–39/2021

Characteristics	Value	Negative controls; n = 668		COVID‐19 cases; n = 1104		*P* value
Age	Median, years [IQR]	81	[70–87]	77	[56–87]	0.005
		N	%	N	%	
Age group	20–29 years	6	0.9	38	3.4	
	30–39 years	7	1.0	67	6.1	
	40–49 years	15	2.2	71	6.4	
	50–59 years	55	8.2	164	14.9	
	60–69 years	79	11.8	126	11.4	
	70–79 years	148	22.2	123	11.1	
	80+ years	358	53.6	515	46.6	0.000
Sex	Male	375	56.1	597	54.1	
	Female	293	43.9	507	45.9	0.398
Presence of chronic condition (one or more)		639	96.1	581	53.4	0.000
	Hypertension	428	64.5	389	35.4	0.000
	Cardiovascular disease	312	47.1	225	20.5	0.000
	Respiratory (incl. asthma)	294	47.3	133	13.1	0.000
	Metabolic (incl. diabetes)	328	53.3	251	24.6	0.000
	Liver disease	28	4.7	35	3.5	0.241
	Renal disease	116	19.3	93	9.3	0.000
	Immunosuppression	63	10.4	38	3.8	0.000
	Other chronic conditions	364	60.6	299	30.1	0.000
Pneumonia		309	51.4	623	84.5	0.000
Mechanical ventilation		5	1.1	40	7.7	0.000
ICU admission		15	2.4	95	10.1	0.000
Death in hospital		59	9.9	157	18.2	0.000
Number of admissions in the last year	None	65	28.1	53	18.5	
	One or two	143	61.9	217	75.9	
	More than two	23	10.0	16	5.6	0.003
History of a previous positive SARS‐CoV‐2 test	No	468	84.3	527	85.3	
	Yes	87	15.7	91	14.7	0.650
Type of SARS‐CoV‐2 test	RT‐PCR	493	97.2	826	75.4	
	Rapid antigen test	14	2.8	269	24.6	0.000
COVID‐19 vaccination status	Unvaccinated	191	28.6	811	73.5	
	Complete vaccination	477	71.4	293	26.5	0.000
Vaccine products (complete vaccination)	Comirnaty	416	87.2	235	80.2	
	Spikevax	24	5.0	14	4.8	
	Janssen	16	3.4	33	11.3	
	Vaxzevria	14	2.9	11	3.8	
	Curevac	5	1.0	0	0.0	
	Comirnaty/Spikevax	1	0.2	0	0.0	
	Comirnaty/Vaxzevria	1	0.2	0	0.0	0.001

PCR was the most commonly used diagnostic test, although rapid antigen test was also used for case confirmation (25%). Rapid tests were less frequently used among controls (3%), in line with the protocol recommendation of a PCR test for confirmation if the initial rapid antigen test was negative.

A total of 71% of controls had received complete COVID‐19 vaccination, compared with 27% of cases. Among those fully vaccinated, Comirnaty (Pfizer/BioNTech BNT162b2) was the most commonly used vaccine in cases (80%) and controls (87%), followed by Janssen (COVID‐19 Vaccine Janssen, Ad26.cov2.s) (11% cases; 3% controls), Spikevax (COVID‐19 Vaccine Moderna, mRNA‐1273) (5% in both cases and controls), and Vaxzevria (AstraZeneca ChAdOx1‐S) (4% cases; 3% controls) (Tables 1 and S2).

### 3.2 VE by age group

**FIGURE 3:**
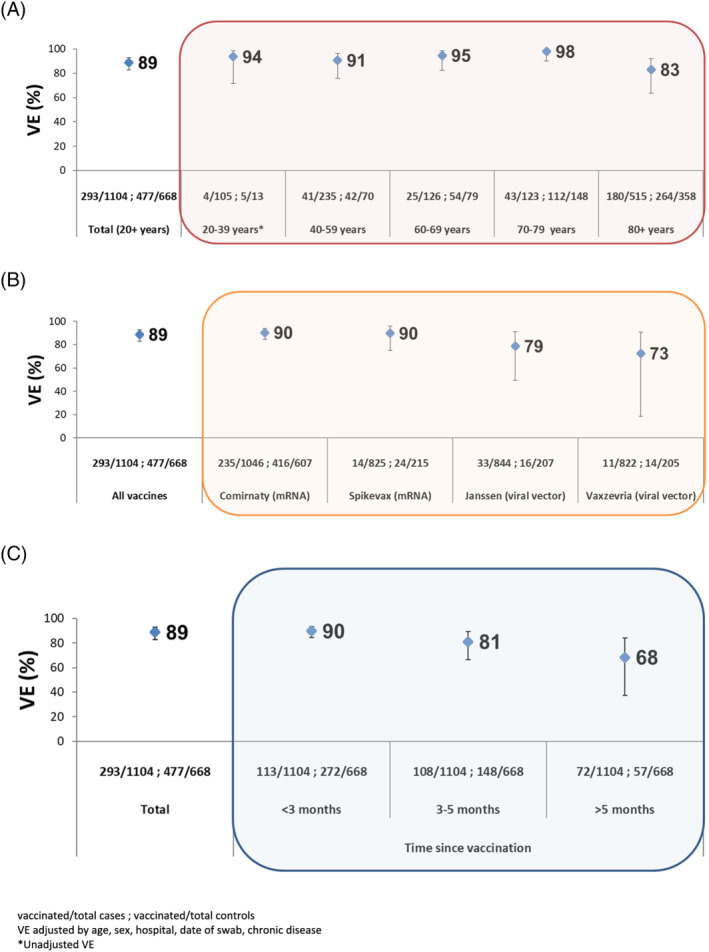
COVID‐19 VE against SARI hospitalization confirmed with COVID‐19, by (A) age group, (B) vaccine type, and (C) time since vaccination, Spanish SARI sentinel surveillance, Weeks 1–39/2021

The overall adjusted VE against COVID‐19 hospitalization was 89% (95% CI: 83–93) among SARI patients aged 20 and older. The VE was 94% (95% CI: 72–99), 91% (95% CI: 76–96), 95% (95% CI: 83–98), 98% (95% CI: 90–100), and 83% (95% CI: 63–92) for those aged 20–39, 40–59, 60–69, 70–79, and 80 years and older, respectively (Figure 3A). For the Comirnaty vaccine only, we observed similar results by age group (Table S3).

### 3.3 VE by vaccine type

By vaccine type, mRNA vaccines (Comirnaty and Spikevax) showed higher VE against COVID‐19 hospitalization than viral vector vaccines (Janssen and Vaxzevria) among those aged 20 years and older. VE was 90% (95% CI: 85–94) for Comirnaty, 90% (95% CI: 75–96) for Spikevax, 79% (95% CI: 49–91) for Janssen vaccine, and 73% (95% CI: 19–91) for Vaxzevria (Figure 3B).

Compared with mRNA vaccines, Janssen VE was lower among those aged 20 to 59 years, and Vaxzevria and Janssen VE were lower among those aged 60 years, although precision is low in these analyses due to the small sample size (Table S4).

### 3.4 VE by time since vaccination

Among those aged 20 and older, VE for all vaccines against COVID‐19 hospitalization by months between vaccination and onset of symptoms was 90% (95% CI: 84–93) at <3 months, 81% (95% CI: 66–89) at 3–5 months, and 68% (95% CI: 38–84) at ≥5 months (Figure 3C). When stratifying by age only for mRNA vaccines, adjusted VE decreased over time among those aged 80 years and older, from 86% (95% CI: 70–79) at <3 months to 48% (95% CI: −51–82) at ≥5 months (Table 2). In younger age groups, VE was maintained over time, although sample size was very small for some estimates (<10 vaccinated cases or controls).

**TABLE 2:** Effectiveness of complete vaccination against COVID‐19 hospitalization among SARI patients, by time since vaccination and vaccine product, Spanish SARI sentinel surveillance, Weeks 1–39/2021

Analysis by time since vaccination; mRNA vaccines (Comirnaty + Spikevax)			
Brand, age group, and time since vaccination	Cases/controls	Crude VE (95% CI)	Adjusted VE (95% CI)^a^
mRNA vaccines, age 20–59 years			
Unvaccinated	295/36		
Vaccinated <3 months	11/29	95 (90–98)	95 (82–98)
Vaccinated 3–5 months	6/4	82 (32–95)	73 (−81 to 96)
Vaccinated >5 months	5/6	90 (65–97)	91 (50–98)
mRNA vaccines, age 60–69 years			
Unvaccinated	101/25		
Vaccinated <3 months	6/21	93 (81–97)	97 (87–99)
Vaccinated 3–5 months	3/9	92 (67–98)	92 (2–99)
Vaccinated >5 months	2/4	88 (29–98)	96 (47–100)
mRNA vaccines, age 70–79 years			
Unvaccinated	80/36		
Vaccinated <3 months	21/63	85 (72–92)	98 (90–100)
Vaccinated 3–5 months	11/40	88 (73–94)	98 (85–100)
Vaccinated >5 months	9/4	−1 (−250 to 71)	91 (24–99)
mRNA vaccines, age 80 + years			
Unvaccinated	335/94		
Vaccinated <3 months	38/126	92 (87–94)	86 (70–94)
Vaccinated 3–5 months	81/91	75 (64–83)	69 (20–88)
Vaccinated >5 months	56/43	63 (42–77)	48 (−51 to 82)

^a^ Adjusted by age, sex, hospital, swab date, and presence of chronic disease.

### 3.5 Characteristics of Alpha and Delta hospitalizations

Among cases with sequencing data notified through SARI sentinel surveillance, 35 were Alpha cases, swabbed between Weeks 7 and 32, and 71 were Delta cases, swabbed between Weeks 27 and 39. Severe outcomes like pneumonia, mechanical ventilation, ICU admission, and death were more frequent among Alpha than Delta cases, and no differences were observed in underlying chronic conditions (Table 3).

**TABLE 3:** Characteristics of SARI controls and cases (n = 642) included in the analysis of COVID‐19 VE by period of Alpha and Delta SARS‐CoV‐2 variant circulation, Spanish SARI sentinel surveillance, Weeks 7–39/2021

	SARS‐CoV‐2 Alpha N = 35		SARS‐CoV‐2 Delta N = 71		*P* value
	N	%	N	%	
Age group					
20–29 years	1	2.9	1	1.4	
30–39 years	3	8.6	10	14.1	
40–49 years	1	2.9	6	8.5	
50–59 years	8	22.9	11	15.5	
60–69 years	6	17.1	12	16.9	
70–79 years	8	22.9	4	5.6	
80+ years	8	22.9	27	38.0	<0.001
Chronic condition					
One or more	28	82.4	59	84.3	0.803
Hypertension	16	45.7	33	46.5	0.941
Heart disease	8	22.9	20	28.1	0.560
Respiratory disease	7	33.3	13	20.3	0.222
Diabetes	8	40.0	24	38.1	0.879
Liver disease	3	17.7	6	9.1	0.312
Renal disease	3	16.7	8	12.7	0.665
Immunosupression	3	18.8	9	14.1	0.639
Other chronic conditions	9	50.0	31	50.0	1.000
Severity					
Pneumonia	33	97.1	50	78.1	0.013
Mechanical ventilation	5	35.7	2	4.7	0.002
ICU admission	7	21.2	5	7.8	0.058
Death in hospital	8	36.4	6	9.4	0.003

### 3.6 VE against Alpha and Delta

Overall VE results in the Alpha and Delta circulation periods were similar (85% [95% CI: 72–92] and 86% [95% CI: 74–92]). Variant‐specific VE was slightly higher against Alpha (97% [95% CI: 84–100]) than Delta (88% [95% CI: 73–95]) (Table 4).

**TABLE 4:** Effectiveness of complete vaccination against COVID‐19 hospitalization among SARI patients, during SARS‐CoV‐2 Alpha and Delta circulation period, and against hospitalization with confirmed Alpha and Delta SARS‐CoV‐2, Spanish SARI sentinel surveillance, Weeks 1–39/2021

Period included and age group	Vaccinated/total cases; vaccinated/total controls	Crude VE (95% CI)	Adjusted VE (95% CI)
Alpha circulation period, Weeks 01/2021–26/2021			
Total (20+ years)	32/578; 172/339	94 (91–96)	85 (72–92)^a^
VE against confirmed Alpha hospitalization, Weeks 7–32/2021			
Total (20+ years)	4/35; 252/370	94 (82–98)	97 (84–100)^b^
Delta circulation period, Weeks 27–39/2021			
Total (20+ years)	261/526; 305/329	92 (88–95)	86 (74–92)^a^
VE against confirmed Delta hospitalization, Weeks 27–39/2021			
Total (20+ years)	39/71; 276/294	92 (85–96)	88 (73–95)^b^

^a^ Adjusted by age, sex, hospital, swab date, and presence of chronic disease.

^b^ Adjusted by age, sex, hospital, month of swab, and presence of chronic disease.

## 4 DISCUSSION

We have used a test‐negative design with information obtained from SARI surveillance to estimate vaccine protection against COVID‐19 hospitalization, which is essential for the evaluation of the impact of COVID‐19 vaccination programs. Our results show high VE of 89% overall between January 1 and September 30, 2021, in persons aged 20 and older, fully vaccinated with any vaccine brand. Protection was higher for mRNA vaccines, and lower for those 80 or older, who also showed a decline in VE after 3 months of completing vaccination, with a further decrease after 5 months. When restricting to mRNA vaccines, the decrease of VE by time since vaccination was only evident in the group over 80, although confidence intervals are wide. We found no differences between periods with circulation of Alpha or Delta SARS‐CoV‐2 variants, although variant‐specific VE was slightly higher against Alpha.

The overall VE estimate is lower than the one estimated soon after the implementation of the vaccination program in Israel,^11^, ^12^, ^13^ Canada,^14^ the United States,^15^, ^16^, ^17^ the United Kingdom,^18^, ^19^ and Spain.^20^, ^21^ However, it approaches more recent estimates, especially from studies including periods with circulation of Delta variant and longer follow‐up time.^22^, ^23^, ^24^, ^25^ Of note, our study included a relatively old population compared with other studies in the literature, which certainly plays a role in the waning observed in those aged 80 and older. Studies analyzing older populations have reached similar estimates. A study in US veterans^23^ found, between February and August 21, a VE of 87% (95% CI: 80% to 91%) against hospitalization overall and of 80% (95% CI: 68% to 87%) for 65 or older versus 95% (95% CI: 89% to 98%) for 18–64 years, similar in the periods of Alpha or Delta dominance. In Portugal,^24^ a registry‐based study found lower VE in people ≥80 years (95% CI: 82%; 72% to 89%) compared with 65–79 years (94%; 95% CI: 88% to 97%). In the United Kingdom,^26^ a test‐negative study of patients admitted to hospital up to February 2021 found a VE of 89% (95% CI: 85% to 93%) in patients aged ≥80 years.

Our results point to a lower VE against COVID‐19 hospitalization in those fully vaccinated with Janssen or Vaxzevria, compared with Spikevax or Comirnaty vaccines, although confidence intervals are wide, especially for Vaxzevria. Analysis in 20–59 and 60–69 age groups showed lower VE for Janssen than for mRNA vaccines, although sample size was insufficient to confirm differences of vaccine protection by vaccine type in different age groups. A lower VE for Janssen, although not for Vaxzevria, had been previously pointed out. In a study in Spain,^20^ Janssen had a VE of 86% versus 97% to 98% for mRNA or Vaxzevria vaccines, and in Navarre,^27^ VE was lower for Janssen (74%; 95% CI: 43% to 88%), but not for Vaxzevria (95%; 95% CI: 79% to 99%), compared with Spikevax (98%; 95% CI: 82% to 100%) or Comirnaty (93%; 95% CI: 88% to 96%). Also, in the United States,^28^ VE for Janssen was 71% (95% CI: 56% to 81%) compared with Spikevax 93% (95% CI: 91% to 95%) or Comirnaty 88% (95% CI: 86% to 91%).

Waning of immunity in the group over 80 years of age, who make up the majority of our study population, is the main driver of the overall decrease. There is great interest to disentangle the relative contribution of waning of immunity and the expansion of Delta variant in explaining increases in transmission experienced in many countries in June and July 2021. Fortunately, waning is mostly found for outcomes of infection^25^, ^29^, ^30^, ^31^, ^32^, ^33^, ^34^, ^35^, ^36^ while evidence of waning of protection against severe infection is less consistent. Our results show protection remained high 5 months after vaccination in persons under 80, in accordance with evidence from randomized clinical trials up to March. These studies, before the Delta variant, found efficacy against severe infection remained at 97% (95% CI: 80% to 100%) and 98% (95% CI: 93% to 100%) after 6 months of randomization to Comirnaty or Spikevax vaccines, respectively.^30^, ^37^ Regarding observational studies in the general population in the United States, VE within 1 month after full vaccination with Pfizer was 87% and 88% after 5 months,^29^ and in New York between May and July, VE was relatively stable, ranging from 89.5% to 95.1%.^25^ In contrast, other studies have found a decrease in protection with time since vaccination, at similar or longer follow‐up times than our study and more generally in all age groups. In a study^28^ in the United States between March and August 2021, in a population with a median age of 58, VE for Pfizer decreased from 91% (95% CI: 88–93%) between 14 and 120 days post‐vaccination to 77% (95% CI: 67–84%) if >120 days post‐vaccination, while VE for Spikevax remained high. As in our study, waning immunity was age dependent in the United Kingdom,^32^ where Delta‐specific VE decreased from 98% (95% CI: 98% to 99%) in Weeks 2–9 after full vaccination with Comirnaty, to 93% (95% CI: 90% to 95%) beyond 20 weeks, being more pronounced for the age group ≥65 (down to 91%). For Vaxzevria vaccine, the decrease was even greater, from 95% (95% CI: 95% to 96%) to 77% (95% CI: 70% to 82%). We were not able to assess differences by time since vaccination, according to vaccine type, because 85% of cases and 92% of controls in our study had been fully vaccinated with mRNA vaccines.

Finally, regarding a potential decrease in protection due to the emergence of the Delta variant, as in our study, most studies have not found differences in VE against hospitalization in the Alpha or Delta dominance periods despite reduced protection against infection.^23^, ^38^, ^39^, ^40^ In our study, only alpha‐specific VE resulted higher than Delta‐specific VE, although in the alpha period, sequencing was less systematic and this could bias the comparison. However, a study in the Netherlands^38^ using aggregated data found similar VE against hospitalization in the Alpha and Delta periods (94% and 95%), with no differences by age groups or time since vaccination (up to 20 weeks).

Our study has several limitations. First, adjusted VE estimates by time since vaccination might be affected by sparse data, mainly in subgroups under 70 years of age vaccinated more than 3 months prior. The test‐negative design has been widely used for influenza VE, among others, within the I‐MOVE network.^41^, ^42^ Because our study is based on SARI surveillance data, it is likely affected by heterogeneity in data collection of SARI admissions among participating hospitals. These data quality issues are inherent to routine epidemiological surveillance particularly during the first weeks of implementation. However, we have demonstrated that the new SiVIRA surveillance system in Spain was able to achieve two of its objectives in the first season after implementation: to monitor severe clinical episodes caused by SARS‐CoV‐2 on a weekly basis,^7^ while monitoring in real‐time COVID‐19 VE. Low compliance in some key variables, such as SARS‐CoV‐2 genetic variant, results in a low sample size for some specific analyses. The consolidation of the SARI surveillance system in later seasons will likely improve homogeneity of data reporting between hospitals and the availability of timely SARS‐CoV‐2 and influenza sequencing data.

## 5 CONCLUSION

In summary, surveillance data from the first season of the SiVIRA hospital network demonstrate the usefulness of sentinel syndromic surveillance systems to describe clinical and epidemiological characteristics of SARI hospitalizations and to monitor the circulation of SARS‐CoV‐2, influenza, and other respiratory viruses, while also providing data to measure the effectiveness of vaccination in the population under surveillance. Our study adds to the evidence of waning of protection against severe COVID‐19 with time since vaccination in those 80 years or older, but with no substantial differences between SARS‐CoV‐2 variants (Alpha or Delta). In addition, this study provides more data on the higher effectiveness of mRNA vaccines compared with Janssen or Vaxzevria. Our results endorse the policy, already approved in Spain,^43^ of administering additional doses, particularly in the population over 80.

## CONFLICT OF INTEREST

The authors report no conflict of interest.

## AUTHOR CONTRIBUTIONS

**Clara Mazagatos:** Conceptualization; formal analysis; data curation; investigation; methodology; writing‐original draft; writing‐review and editing. **Concepción Delgado‐Sanz:** Data curation; writing‐review and editing. **Susana Monge:** Conceptualization; methodology; writing‐original draft; writing‐review and editing. **Francisco Pozo:** Investigation; writing‐review and editing. **Jesús Oliva:** Data curation; writing‐review and editing. **Virginia Sandonis:** Investigation; writing‐review and editing. **Ana Gandarillas:** Investigation; writing‐review and editing. **Carmen Quiñones‐Rubio:** Investigation; writing‐review and editing. **Cristina Ruiz‐Sopeña:** Investigation; writing‐review and editing. **Virtudes Gallardo‐García:** Investigation; writing‐review and editing. **Luca Basile:** Investigation; writing‐review and editing. **María Isabel Barranco‐Boada:** Investigation; writing‐review and editing. **Olga Hidalgo‐Pardo:** Investigation; writing‐review and editing. **Olalla Vazquez‐Cancela:** Investigation; writing‐review and editing. **Miriam García‐Vázquez:** Investigation; writing‐review and editing. **Amelia Fernández‐Sierra:** Investigation; writing‐review and editing. **Ana Milagro‐Beamonte:** Investigation; writing‐review and editing. **María Ordobás:** Investigation; writing‐review and editing. **Eva Martínez‐Ochoa:** Investigation; writing‐review and editing. **Socorro Fernández‐Arribas:** Investigation; writing‐review and editing. **Nicola Lorusso:** Investigation; writing‐review and editing. **Ana Martínez:** Investigation; writing‐review and editing. **Ana García‐Fulgueiras:** Investigation; writing‐review and editing. **Bartolomé Sastre‐Palou:** Investigation; writing‐review and editing. **Isabel Losada‐Castillo:** Investigation; writing‐review and editing. **Silvia Martínez‐Cuenca:** Investigation; writing‐review and editing. **Mar Rodríguez‐del Águila:** Investigation; writing‐review and editing. **Miriam Latorre:** Investigation; writing‐review and editing. **Amparo Larrauri:** Conceptualization; funding acquisition; methodology; supervision; writing‐original draft; writing‐review and editing. **SARI Surveillance VE group in Spain**: Investigation; writing‐review and editing.

## THE SARI SURVEILLANCE VE GROUP IN SPAIN

Irene Pedrosa Corral (Servicio de Microbiología, Hospital Universitario Virgen de las Nieves; Instituto de Investigación Biosanitaria, Granada); Elvira García Cueva (Servicio Medicina Preventiva. Hospital Universitario Virgen de las Nieves, Granada); Cristina Fernández Jiménez (Vigilancia Epidemiológica, Dirección General de Salud Pública, Departamento de Sanidad, Gobierno de Aragón); Clara Berrozpe (Servicio de Medicina Preventiva, Hospital Universitario Miguel Servet, Zaragoza); Pablo A. Fraile‐Ribot, Carla López‐Causapé (Servicio de Microbiología, Hospital Universitario Son Espases; Instituto de Investigación Sanitaria Illes Balears (IdISBa); CIBERINFEC); María Dolores Garcia Arcal (Servicio de Medicina Preventiva, Hospital Universitario de Burgos); María Isabel Andrés Franch (Servicio de Microbiología, Hospital Universitario de Burgos); Cristina Hernán García (Servicio de Medicina Preventiva, Hospital Clínico Universitario de Valladolid); Silvia Rojo Rello (Servicio de Microbiología, Hospital Clínico Universitario de Valladolid); Aleix Soler Garcia (Servicio de Pediatría, Hospital Sant Joan de Déu Barcelona); Ana Vilella (Servicio de Medicina Preventiva, Hospital Clínic de Barcelona); María Ángeles Marcos, Mar Mosquera (Laboratorio de Microbiología, Hospital Clínic de Barcelona); Antonio Aguilera, María Luisa Pérez del Molino (Servicio de Microbiología, Complejo Hospitalario Universitario de Santiago); Ana Blanco Ferreiro (Servicio de Medicina Preventiva, Complejo Hospitalario Universitario de Santiago, Santiago de Compostela); Ramón Domenech (Subdirección General de Epidemiología, Dirección General de Salud Pública, Comunidad de Madrid; Fundación para la Investigación y la Innovación Biosanitaria de Atención Primaria, Comunidad de Madrid); Nicolás García‐Arenzana Les (Servicio de Medicina Preventiva, Hospital Universitario La Paz); Iker Falces Romero (Laboratorio de Microbiología, Hospital Universitario La Paz; CIBERINFEC); Beatriz Nieto Pereda (Servicio de Medicina Preventiva, Hospital Universitario Gregorio Marañón); Patricia Muñoz (Laboratorio de Microbiología, Hospital Universitario Gregorio Marañón; Instituto de Investigación Sanitaria Hospital Gregorio Marañón; Departamento de Medicina, Facultad de Medicina, Universidad Complutense de Madrid; CIBERES); Amaranta Mcgee (Servicio de Medicina Preventiva, Hospital Universitario Ramón y Cajal); Laura Martínez García (Laboratorio de Microbiología, Hospital Universitario Ramón y Cajal); Antonio Moreno Docon (Servicio de Microbiología, Hospital Clínico Universitario Virgen de Arrixaca); Noemí Zapata Castaño, Alberto Torres Cantero (Servicio de Medicina Preventiva, Hospital Clínico Universitario Virgen de Arrixaca); Ana Carmen Ibañez Perez (Servicio de Epidemiología y Prevención Sanitaria, Dirección General de Salud Pública, Consumo y Cuidados, La Rioja); Miriam Blasco Alberdi (Laboratorio de Microbiología, Hospital San Pedro de Logroño); Inmaculada Casas Flecha (National Centre for Microbiology, Institute of Health Carlos III, Madrid, Spain; CIBERESP); Sonia Vázquez Morón, María de la Montaña Iglesias Caballero (National Centre for Microbiology, Institute of Health Carlos III, Madrid, Spain).

## Supporting information




**Data S1.** Supporting Information

## Data Availability

Data access policy within the National Epidemiological Surveillance Network (RENAVE) is similar to that of other Public Health Agencies, such as the European Centre for Disease Control. The RENAVE, managed and maintained by the National Centre of Epidemiology, has the mandate to collect, analyze, and disseminate surveillance data on infectious diseases in Spain. There is no direct access to the RENAVE database, but data are available upon request.
