# Supplementary material for: COVID‐19 vaccine effectiveness against hospitalization due to SARS‐CoV‐2: A test‐negative design study based on Severe Acute Respiratory Infection (SARI) sentinel surveillance in Spain
Source: Influenza Other Respir Viruses. 2022 Jul 26;16(6):1014–25. doi: 10.1111/irv.13026 (PMC9350393; doi:10.1111/irv.13026)
Supplement: Supplementary file 1 — Data S1. Supporting Information [file IRV-16-1014-s001.docx]

# COVID-19 vaccine effectiveness against hospitalization due to SARS-CoV-2: A test negative design study based on SARI sentinel surveillance in Spain

**SUPPLEMENTARY MATERIAL**

**Appendix 1. Methodological information**

**SiVIRA sentinel surveillance case definition for SARI:**

A SARI case is defined as a patient presenting with:

- A respiratory infection with at least one of the following symptoms: Cough, shortness of breath, sore throat, runny nose, with or without fever
- Which is acute, with onset in the last 10 days
- And severe, requiring hospitalization

**Definitions of COVID-19 vaccination status**

Complete vaccination was defined based on the vaccine brand, and considering the number of doses, the days between doses and days after last dose recommended for each vaccine, according to product specifications:

- For Comirnaty vaccine: Two vaccine-doses separated by at least 19 days and after 7 days of the second dose, or only one dose in a person with previous documented SARS-CoV-2 infection, 7 days after that single dose.
- For Spikevax vaccine: Two vaccine-doses separated by at least 25 days and after 14 days of the second dose, or only one dose in a person with previous documented SARS-CoV-2 infection, 14 days after that single dose.
- For Vaxzevria vaccine: Two vaccine-doses separated by at least 21 days and after 14 days of the second dose, or only one dose in a person with previous documented SARS-CoV-2 infection, 14 days after that single dose.
- For Janssen vaccine: One vaccine-dose, after 14 days of this single dose.

**Appendix 2. Additional tables and figures.**

Table S1. Start date of inclusion of patients eligible for COVID-19 vaccination, according to their age group.

| **Target group** | **Start date for inclusion** |
| --- | --- |
| 80 years and older | 01/01/2021 |
| 75 years and older | 07/03/2021 |
| 70 years and older | 01/04/2021 |
| 60 years and older | 07/04/2021 |
| 50 years and older | 07/04/2021 |
| 40 years and older | 20/06/2021 |
| 30 years and older | 01/07/2021 |
| 20 years and older | 10/07/2021 |

Table S2. Number and proportion of SARI patients by age group and vaccine type included in the VE study, Spanish SARI sentinel surveillance, weeks 1-39/2021

|  | **20-39 years** | | **40-59 years** | | **60-69 years** | | **70-79 years** | | **80+ years** | | **Total**  **(20+ years)** | |
| --- | --- | --- | --- | --- | --- | --- | --- | --- | --- | --- | --- | --- |
|  | **N** | **%** | **N** | **%** | **N** | **%** | **N** | **%** | **N** | **%** | **N** | **%** |
| Unvaccinated | 109 | 92.4 | 222 | 72.8 | 126 | 61.5 | 116 | 42.8 | 429 | 49.1 | 1002 | 56.5 |
| Comirnaty | 6 | 5.1 | 46 | 15.1 | 33 | 16.1 | 139 | 51.3 | 427 | 48.9 | 651 | 36.7 |
| Spikevax | 0 | 0.0 | 9 | 3.0 | 12 | 5.9 | 9 | 3.3 | 8 | 0.9 | 38 | 2.1 |
| Janssen | 3 | 2.5 | 25 | 8.2 | 10 | 4.9 | 4 | 1.5 | 7 | 0.8 | 49 | 2.8 |
| Vaxzevria | 0 | 0.0 | 3 | 1.0 | 21 | 10.2 | 1 | 0.4 | 0 | 0.0 | 25 | 1.4 |
| Curevac | 0 | 0.0 | 0 | 0.0 | 2 | 1.0 | 2 | 0.7 | 1 | 0.1 | 5 | 0.3 |
| Comirnaty/Spikevax | 0 | 0.0 | 0 | 0.0 | 0 | 0.0 | 0 | 0.0 | 1 | 0.1 | 1 | 0.1 |
| Comirnaty/Vaxzevria | 0 | 0.0 | 0 | 0.0 | 1 | 0.5 | 0 | 0.0 | 0 | 0.0 | 1 | 0.1 |
| Total | 118 | 100 | 305 | 100 | 205 | 100 | 271 | 100 | 873 | 100 | 1772 | 100 |

Table S3. Effectiveness of complete vaccination with Comirnaty against COVID-19 hospitalization among SARI patients, by age group, Spanish SARI sentinel surveillance, weeks 1-39/2021

| **Analysis by age group; Comirnaty** | | | |
| --- | --- | --- | --- |
| **Brand, age group and time since vaccination** | **Vaccinated/total cases; vaccinated/total controls** | **Crude VE (95% CI)** | **Adjusted VE (95% CI)^a^** |
| **Comirnaty vaccine** | | | |
| Age 20-59 years | 18/313 ; 34/70 | 94 (87–97) | 90 (71–96) |
| Age 60-69 years | 6/107 ; 27/52 | 94 (85–98) | 98 (87–100) |
| Age 70-79 years | 39/119 ; 100/136 | 82 (70–90) | 97 (87–99) |
| Age 80+ years | 172/507 ; 255/349 | 81 (74–86) | 72 (40–87) |

^a^ Adjusted by age, sex, hospital, swab date, presence of chronic disease

Table S4. Effectiveness of complete vaccination against COVID-19 hospitalization among SARI patients aged 20-59 and 60-69 years, by vaccine product, Spanish SARI sentinel surveillance, weeks 1-39/2021

| **Analysis by age group and vaccine product** | | | |
| --- | --- | --- | --- |
| **Brand, age group and time since vaccination** | **Vaccinated/total cases; vaccinated/total controls** | **Crude VE (95% CI)** | **Adjusted VE (95% CI)^a^** |
| **Age 20-59 years** | | | |
| mRNA (Comirnaty + Spikevax) | 22/317 ; 39/75 | 93 (87–96) | 92 (78–97) |
| Janssen | 20/315 ; 8/44 | 69 (26–87) | 71 (-3–92) |
| **Age 60-69 years** |  |  |  |
| mRNA (Comirnaty + Spikevax) | 11/112 ; 34/59 | 92 (82–96) | 97 (86–99) |
| Janssen | 6/107 ; 4/29 | 63 (-42–90) | 82 (-78–98) |
| Vaxzevria | 8/109 ; 13/38 | 85 (59–94) | 89 (25–98) |

^a^ Adjusted by age, sex, hospital, swab date, presence of chronic disease
